# DCIS Progression and the Tumor Microenvironment: Molecular Insights and Prognostic Challenges

**DOI:** 10.3390/cancers17121925

**Published:** 2025-06-10

**Authors:** Karolina Prajzendanc

**Affiliations:** Department of Genetics and Pathology, International Hereditary Cancer Center, Pomeranian Medical University, 71-252 Szczecin, Poland; karolina.prajzendanc@pum.edu.pl

**Keywords:** ductal carcinoma in situ, DCIS progression, tumor microenvironment

## Abstract

Ductal carcinoma in situ (DCIS) is the most common form of non-invasive breast cancer and a recognized precursor to invasive ductal carcinoma (IDC). Although DCIS itself is confined to the milk duct and not immediately life-threatening, its potential for progression to invasive disease necessitates careful clinical management. The increased detection of DCIS due to advancements in imaging and widespread screening programs has raised critical questions regarding its classification, prognosis, and optimal treatment strategies. While most cases exhibit indolent behavior, others harbor molecular characteristics that drive malignant transformation. A key challenge lies in distinguishing low-risk DCIS, which may never progress, from aggressive cases requiring intervention. Tumor microenvironment dynamics, immune cell infiltration, and molecular alterations, including hormone receptor (HR) status, human epidermal growth factor 2 (HER2) expression, and genetic mutations, play crucial roles in determining disease trajectory. This review explores the biological and molecular mechanisms underlying DCIS progression, with an emphasis on myoepithelial cells, tumor-infiltrating lymphocytes, and microenvironmental factors. By integrating recent findings, this article aims to refine risk stratification approaches and guide future strategies for personalized DCIS management. Improved prognostic biomarkers and targeted therapeutic interventions could help optimize treatment decisions, balancing the need for effective cancer prevention while minimizing overtreatment in low-risk patients.

## Simple Summary

Ductal carcinoma in situ is a prevalent non-invasive form of breast cancer that, while confined to the milk ducts, carries the potential to progress to invasive disease. With increased detection due to advanced imaging and widespread screening, there is growing concern about the ability to accurately predict which cases are likely to become aggressive. This review aims to deepen the understanding of the biological mechanisms that influence DCIS progression, focusing on the roles of the tumor microenvironment, immune response, and molecular alterations. By synthesizing the contribution of myoepithelial cells, tumor-infiltrating lymphocytes, and key molecular markers, the review seeks to enhance risk stratification methods. These insights could support the development of more precise, personalized treatment strategies that effectively prevent cancer progression while minimizing unnecessary interventions in patients with low-risk DCIS.

## 1. Introduction

DCIS is the most common form of non-invasive breast cancer, representing approximately 15–25% of all breast cancers detected through screening mammography [1,2,3]. DCIS is characterized by the presence of abnormal epithelial cells confined to the milk ducts of the breast and is considered a precancerous lesion within the spectrum of breast cancer progression. While DCIS itself is non-invasive and not immediately life-threatening, if left untreated, it has the potential to develop into IDC, which can metastasize and significantly affect patient outcomes [4].

Advances in imaging techniques and the widespread adoption of breast cancer screening programs have significantly increased the detection of DCIS over the past few decades [5]. However, this has also introduced challenges in distinguishing indolent cases from those with aggressive potential, resulting in many questions and discussions about optimal management and treatment strategies [6,7]. Current approaches typically involve surgery, either breast-conserving or mastectomy, often complemented by radiation therapy or hormonal treatments, depending on individual risk factors and tumor characteristics [8].

There is an ongoing debate in the medical community about whether DCIS should be classified as cancer [9]. Some argue that since it is treated similarly to small invasive breast cancer, there is no need to label it differently and risk confusing patients [7]. This perspective seems logical, but the real question is whether DCIS truly requires the same treatment approach as invasive breast cancer in all cases. Long-term studies suggest that in up to 70% of cases, DCIS does not progress to invasive disease [4,10,11]. Regardless of terminology, the critical challenge remains distinguishing “safe” cases from those with malignant potential. While ensuring that high-risk patients receive appropriate treatment is essential, equally important is sparing low-risk individuals from unnecessary interventions and their potential side effects. Together, these challenges highlight the urgent need for improved risk stratification tools and better molecular profiling to optimize DCIS management and reduce both overtreatment and undertreatment.

This review provides a comprehensive overview of DCIS, with a particular focus on the molecular features associated with its progression to invasive cancer. By synthesizing recent research, it aims to deepen the understanding of DCIS biology and its potential for invasive transformation. Ultimately, this review highlights the need for further research and the implementation of advanced techniques, which hold the potential to uncover previously unknown insights.

## 2. Histology of DCIS

DCIS is characterized by the proliferation of malignant epithelial cells within the milk ducts of the breast, remaining confined by the basement membrane (BM)—a structural barrier that separates the epithelial layer from the surrounding stroma. This containment prevents access to blood vessels and lymphatic channels, thereby inhibiting metastasis. DCIS can remain in situ for years, even for a lifetime [10]. Understanding the biological, molecular, and clinical factors that either maintain this containment or drive progression is essential for developing targeted prevention strategies and optimizing patient outcomes.

Histologically, DCIS exhibits diverse architectural patterns, including comedo, cribriform, papillary, micropapillary, and solid forms [12]. Its grade—classified as low, intermediate, or high—reflects the degree of cellular atypia and mitotic activity, with high-grade lesions demonstrating a greater propensity for progression [11,13]. Low-grade DCIS closely resembles atypical ductal hyperplasia and retains features similar to normal breast cells, while intermediate-grade DCIS shares characteristics with low-grade lesions but exhibits a higher proliferation rate. High-grade DCIS, in contrast, is marked by rapid growth and significant cellular abnormalities and is frequently associated with comedonecrosis and overexpression of molecular markers such as HER2, contributing to its aggressive nature [14]. Patients with high-grade lesions face an elevated risk of progression to IDC and recurrence [11,15,16]. DCIS is most commonly diagnosed as high-grade (42–54%) or intermediate-grade (27–43%), while low-grade cases account for a smaller proportion (14–18%) [5,17]. However, a single DCIS lesion can contain regions of varying grades, complicating the diagnostic process [18]. Another challenge lies in the lack of reproducibility in grading assessments among pathologists [19]. Despite these limitations, nuclear grade—alongside lesion size, margin status, and patient age—remains a key factor in guiding treatment decisions [20,21]. The 10-year risk of progression to IDC is estimated at 12.2% for patients with low- or intermediate-grade DCIS, rising to 17.6% for those with high-grade lesions [11].

## 3. DCIS with Microinvasion

**Figure 1:**
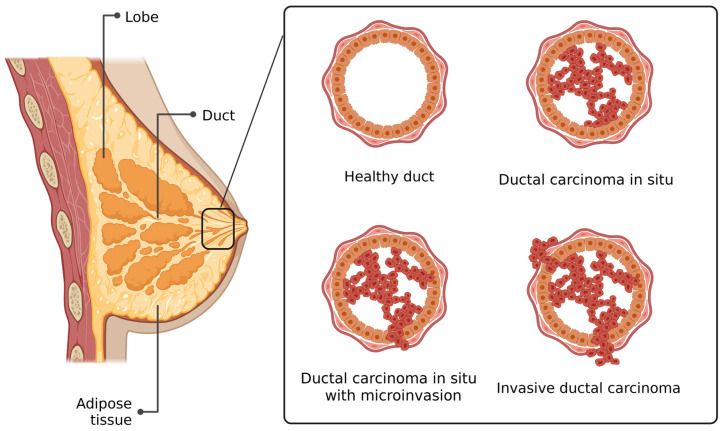
DCIS progression. Progressive stages of breast tissue transformation: starting with normal ductal epithelium, followed by ductal carcinoma in situ (DCIS) characterized by abnormal epithelial proliferation confined within the duct, then DCIS with microinvasion showing early cancer cell escape into the surrounding stroma, and finally, invasive ductal carcinoma (IDC), where malignant cells have fully breached the basement membrane and infiltrated the adjacent tissue.

While DCIS accounts for approximately 20% of newly diagnosed breast cancers, DCIS with microinvasion (DCIS-Mi) constitutes only about 1% of cases [22]. Microinvasion in DCIS is defined as an invasive focus measuring ≤1 mm in diameter, representing an actual, albeit minimal, breach of the basement membrane surrounding the milk duct [23]. DCIS-Mi is considered an intermediate pathological stage between DCIS and IDC [24,25] (Figure 1). Microinvasion may occur as one or multiple foci, but each individual focus must not exceed 1 mm in size [22]. Studies have shown that DCIS-Mi with multiple invasion foci is associated with a higher risk of lymph node metastasis and poorer metastasis-free and overall survival compared to cases with a single invasive focus [24]. Additionally, the presence of multiple invasive foci correlates with an increased risk of local recurrence compared to DCIS-Mi with only one focus [26].

DCIS-Mi more frequently exhibits high nuclear grade compared to pure DCIS [24,26]. Lymph node metastases are observed in 0–13% of DCIS-Mi cases, with a notably higher incidence in those with multiple invasion foci (10.1–15.6%) compared to cases with a single focus (0–2.1%) [22,23,24,25]. In contrast, pure DCIS is associated with a much lower frequency of lymph node metastases, ranging from approximately 0.5% to 1.4%, raising the possibility of misdiagnosis in some cases [23,24]. Tumors with microinvasion are less likely to be estrogen receptor (ER) positive but more frequently exhibit HER2 overexpression and a high Ki-67 proliferation index, features commonly associated with more aggressive cancers [27]. Extensive tumor size, comedo-type architecture, and ER negativity were identified as independent predictors of microinvasion [27]. Clinically, patients with DCIS-Mi have worse 5-year disease-free survival (DFS) than those with pure DCIS but a better prognosis than patients with IDC, with reported DFS rates of 100%, 97%, and 88%, respectively [28].

## 4. Molecular Characteristics of DCIS

Determining hormonal receptor status and the Ki-67 proliferation index are essential steps in characterizing breast cancer, including DCIS, as they form the basis for classifying tumors into four molecular subtypes: Luminal A (HR+ and Ki-67 < 14%), Luminal B (HR+ and Ki-67 ≥ 14%), HER2+ (HR− and HER2+), and basal-like (triple-negative) [23,29,30]. While surrogate immunohistochemical markers are commonly used to classify DCIS into intrinsic subtypes in descriptive studies, it is important to emphasize that this classification lacks clinical and prognostic validation in both pure DCIS and microinvasive cases. Consequently, DCIS subtyping does not yet influence treatment decisions to the same extent as in invasive breast cancer. Nevertheless, it offers valuable prognostic insights, helping to assess tumor aggressiveness, risk of recurrence, and potential for disease progression. Notably, different DCIS subtypes serve as direct precursors to their corresponding invasive cancer subtypes [14,31,32].

The expression of specific receptors and proteins in cancer cells serves as biomarkers that provide insight into prognosis, treatment response, and, most critically in pure DCIS, the likelihood of progression to invasive cancer or recurrence after treatment. Additionally, emerging molecular markers, including gene mutations and epigenetic modifications, are being explored for their potential to refine risk stratification and improve therapeutic decision-making [33].

### 4.1. HER2

HER2 may play a crucial role in the development and progression of DCIS, as its overexpression is more frequently observed in DCIS cells than in IDC [34]. This disproportion is explained by the fact that HER2 overexpression is considered an early event in carcinogenesis, primarily driving proliferation rather than invasion [35]. HER2, a protein encoded by the *ERBB2* gene, is a member of the ERbB family of receptor tyrosine kinases, which regulate cell growth and differentiation [36]. Amplification of the *ERBB2* gene results in up to a 100-fold increase in HER2 receptor expression on the surface of cancer cells compared to normal mammary cells [37]. This overexpression drives excessive signaling, promoting tumor growth, aggressive behavior, and poor prognosis in invasive cases [38]. In DCIS, HER2 overexpression is associated with adverse clinicopathological features, such as larger tumor size, a high Ki-67 index, and the presence of comedonecrosis [39]. Some studies have linked HER2 overexpression in DCIS to an increased risk of recurrence following surgical excision without radiation therapy [40]. In contrast, low HER2 expression in DCIS is associated with lower tumor grade, low Ki-67 levels, and positive hormone receptor status [41].

### 4.2. Germline Mutations

Germline mutations in tumor suppressor genes associated with breast cancer predisposition have been poorly studied in the context of DCIS alone. However, a large study involving over 16,000 DCIS patients identified pathogenic variants in nine breast cancer predisposition genes in 6.5% of cases [42]. Variants in *ATM*, *BRCA1*, *BRCA2*, *CHEK2*, and *PALB2* were significantly associated with over two-fold increased risk of DCIS, with *BRCA2* mutations conferring the highest risk at nearly five-fold. The frequency of high-risk gene mutations (*BRCA1*, *BRCA2*, *PALB2*) is lower in DCIS patients compared to those with IDC, while the prevalence of moderate-risk gene variants (*CHEK2*, *ATM*) is similar in both groups [42]. In another study of 655 DCIS patients, *BRCA2* and *CHEK2* mutations were the most prevalent, occurring at rates of 3.4% and 2.4%, with corresponding odds ratios of 27.96 (95% CI: 6.56–119.26) and 8.08 (95% CI: 2.93–22.05). In contrast, mutations in *PALB2*, *BRCA1*, and *TP53* were less common, with frequencies of 0.9%, 0.6%, and 0.5%, respectively [43]. These results are consistent with a recent Polish study conducted on 564 DCIS cases and 4,702 controls [44]. The frequency of mutations in the *BRCA1* gene was 1.24% among DCIS cases and was associated with an over three-fold increased risk of developing DCIS (OR = 3.27, p = 0.01). *BRCA2* mutations had a similar frequency (1.42% among DCIS cases), with an odds ratio of 11.3 (*p* < 0.0001). These findings underscore the significance of germline mutations in the context of DCIS, supporting their continued use in risk assessment models and preventive strategies. Furthermore, the data highlight the importance of offering genetic testing—particularly for *BRCA1/2* mutations—to all patients diagnosed with DCIS.

### 4.3. Somatic Mutations

The most common somatic mutations detected in DCIS tumor tissues involve *PIK3CA*, *GATA3*, *TP53*, *ERBB2*, and *AKT1* genes [45,46,47,48]. *PIK3CA* mutations are detected in approximately 30% of DCIS, which is similar to DCIS-accompanied IDC or IDC alone [49,50]. However, some studies suggest that *PIK3CA* mutations may be restricted to the DCIS component in cases of DCIS-associated IDC [45]. Additionally, *PIK3CA* mutations have been correlated with positive progesterone receptor (PR) status in DCIS [47]. *TP53* mutations are identified in 15–30% of DCIS cases [51] and are more frequently associated with high-grade, PR-negative tumors that exhibit *ERBB2* amplification [48]. In general, pure DCIS harbors fewer somatic variants compared to DCIS with associated IDC or IDC alone [46,47]. Synchronous DCIS and IDC exhibit high genomic similarity, with some genes, such as *PIK3CA* and *TP53*, demonstrating 100% concordance. However, certain mutations appear to be restricted to the invasive component [45,46,47,52]. Overall, the mutational profiles of DCIS and IDC are highly concordant, suggesting that most genetic alterations arise early in tumorigenesis during the transition from normal breast tissue to DCIS rather than from DCIS to IDC [53]. As a result, only a few genes have been identified as potential drivers of DCIS progression.

## 5. Myoepithelial Markers

**Figure 2:**
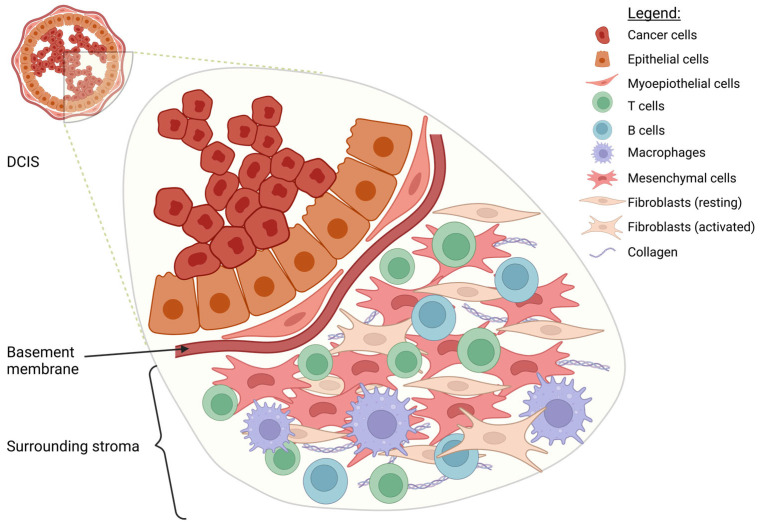
DCIS microenvironment. Surrounding the duct, the stroma reveals a rich mosaic of non-neoplastic cell types contributing to the tumor microenvironment. Immune cells, such as macrophages and lymphocytes (including T and B cells), are dispersed throughout the stroma, indicating active immunological surveillance and potential immunoediting. Fibroblasts, including cancer-associated fibroblasts (CAFs), are embedded within the extracellular matrix among mesenchymal cells. Collagen fibers are prominently visible in the stroma, forming a dense and structured extracellular matrix that provides mechanical support and modulates cell behavior within the DCIS microenvironment. The cellular heterogeneity evident in this microenvironment underscores the dynamic crosstalk between tumor cells and stromal components.

Myoepithelial cells (MECs) are crucial for the proper development and function of the mammary gland and play a vital role in cancer suppression and preventing DCIS progression to invasive cancer [54,55]. Together with the basement membrane, the MEC layer forms a natural border between cancer cells inside the milk duct and the mammary stroma (Figure 2). MECs also produce several tumor-suppressive factors, such as maspin and connexin, which contribute to their protective function [54,56,57]. In addition, MECs express several markers on the surface, which also contribute to suppressor function, and their phenotype has been shown to undergo changes during cancer progression, potentially influencing the transition from DCIS to invasive carcinoma [58,59].

**Figure 3:**
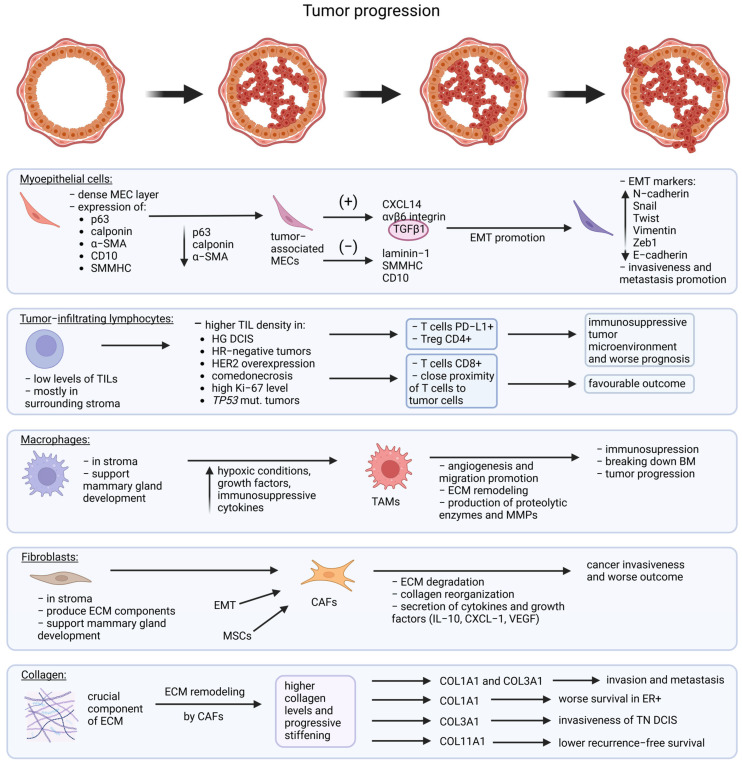
Tumor progression and molecular changes. Dynamic changes in key components of the tumor microenvironment—myoepithelial cells, tumor-infiltrating lymphocytes (TILs), macrophages, fibroblasts, and collagen fibers—from normal ductal epithelium through DCIS and microinvasion to invasive ductal carcinoma are varied and numerous. Myoepithelial Cells: In normal ducts, they form a continuous layer with tumor-suppressive functions. During DCIS, they persist but lose functional integrity. With microinvasion, gaps emerge, and in IDC, they are lost at invasive sites, marking the breakdown of a critical barrier. TILs: Rare in normal tissue, they accumulate in DCIS as an immune response to dysplasia. Over time, they become dysfunctional or exhausted, particularly in invasive stages, despite increased numbers. Macrophages: Initially sparse, they are recruited during DCIS and shift to a tumor-promoting (TAM) phenotype. In IDC, they localize to invasive fronts, secreting factors that support invasion, immune evasion, and angiogenesis. Fibroblasts/CAFs: Normally maintaining ECM balance, fibroblasts activate in DCIS and differentiate into CAFs. These cells promote tumor growth and matrix remodeling, becoming increasingly abundant and interactive with other stromal cells in IDC. Collagen Fibers: Sparse and disorganized in healthy tissue, collagen becomes denser and aligned during progression. In IDC, it forms stiff, linear tracks that facilitate invasion and contribute to therapeutic resistance.

Several key markers, including p63 [60], calponin [61], CD10 [62,63], and T-cell factor 7 (TCF7) [64,65], are considered crucial in inhibiting breast cancer development. Studies by Russell et al. have demonstrated that changes in marker expression can occur even before cancer cells breach the myoepithelial layer [66]. Among the three studied markers, p63 is the first to decrease, followed by calponin, and finally, α-smooth muscle actin (α-SMA). Notably, decreased expression of calponin in MEC has been shown to trigger p63 expression in adjacent cancer cells, suggesting a complex interplay between these proteins during tumor progression [66]. The development of cancer, first in situ, is accompanied by chronic inflammation and interactions between cancer cells and the surrounding microenvironment, which affect the phenotype of both epithelial and myoepithelial cells [67,68]. DCIS-associated MECs exhibit altered gene expression profiles, including reduced levels of tumor-suppressive markers such as laminin-1, smooth muscle myosin heavy chain (SMMHC), CD10, cytokeratin 14 (CK14), p63, or calponin, along with increased expression of oncogenic factors like chemokine (C-X-C motif) ligand 14 (CXCL14) and αvβ6 integrin. These changes contribute to the loss of tumor-suppressive function and the acquisition of pro-oncogenic properties [58,59,69,70]. Additionally, DCIS-associated MECs secret transforming growth factor beta 1 (TFGβ1), which activates the TGFβ/Smads signaling pathway, promoting epithelial–mesenchymal transition (EMT) and further facilitating cancer progression [71] (Figure 3).

Ding et al. conducted a compelling study on breast tissue from healthy women, revealing that phenotypic differences in myoepithelial cells can be observed even in the absence of adjacent tumor cells [72]. The entire cell population analyzed was CD10+, but the authors identified two distinct subgroups based on CD44 expression. Cells expressing CD44 exhibited a more mesenchymal phenotype with higher levels of stem/progenitor cell markers, whereas CD44-negative cells displayed a more epithelial-like phenotype. The most significant differences were observed between *BRCA1* germline mutation carriers and non-carriers. *BRCA1* mutation carriers showed significantly lower expression of key transcription factors, including p63 and TCF7, as well as other myoepithelial markers such as CD10, α-SMA, and CD44. Notably, p63 and TCF7—which typically co-express and co-localize—were much less frequently observed together in *BRCA1* carriers, suggesting that these alterations in myoepithelial cells may contribute to their increased breast cancer risk. In contrast, *BRCA2* mutation carriers displayed normal expression of p63 and TCF7. In patients with DCIS, the majority of CD10+ cells also expressed CD44, reflecting a more mesenchymal phenotype, with lower expression of p63 and TCF7, particularly in high-grade DCIS [72].

Epithelial–mesenchymal transition occurs when epithelial cells gain mesenchymal features, including cytoskeletal reorganization, loss of cell polarity, and disruption of cell junctions, ultimately leading to increased cell motility [73]. EMT is strongly driven by the TGFβ/Smads signaling pathway, which is activated by proteins secreted by cancer cells and DCIS-associated MECs, such as TGFβ1 and αvβ6 integrin [71,74]. During EMT, MECs undergo phenotypic changes, exhibiting increased expression of EMT-associated markers [75]. Higher levels of N-cadherin, Snai1, Twist1, Vimentin, and Zeb1, along with reduced E-cadherin expression, correlate with more progressive disease and are more frequently observed in IDC than DCIS or normal tissue [68]. EMT contributes to invasive cancer progression and metastases, and its induction via the TGFβ/Smads pathway may be a key driver of DCIS transition to invasive carcinoma [73,76].

## 6. Tumor Microenvironment

A milk duct affected by DCIS is surrounded by a stromal microenvironment rich in fibroblasts and immune cells (Figure 2). This stromal compartment plays a crucial role in tumor progression by influencing the immune response, extracellular matrix remodeling, and potential invasion. Notably, the presence and composition of tumor-infiltrating lymphocytes (TILs) within the stroma are of particular interest, as they may affect prognosis and disease progression [77] (Figure 3).

### 6.1. Tumor-Infiltrating Lymphocytes

Due to the presence of an intact myoepithelial layer and basement membrane, TILs are rarely observed within the ducts and, instead, localize in the surrounding stroma [78]. Numerous studies have shown that higher TIL density is associated with high-grade DCIS, HR negativity, HER2 overexpression, comedonecrosis, high Ki-67 level, and a basal-like phenotype [78,79,80,81,82,83]. Additionally, TIL level is significantly elevated in tumors with *TP53* mutations [84,85,86,87] but not in those with *PIK3CA* or *GATA3* mutations [85]. With chronic exposure to tumor antigens, T cells become exhausted, leading to the upregulation of multiple immune checkpoint receptors, including programmed cell death receptor 1 (PD-1) and T cell immunoreceptor with immunoglobulin and ITIM domain (TIGIT), which impair T cell survival and function [88]. Notably, higher stromal TIL density in DCIS correlates with programmed death-ligand 1 (PD-L1) expression on both tumor and immune cells, with PD-L1+ immune cells frequently found in high-grade HR-negative and HER2-positive DCIS [82,85,86]. PD-L1 inhibits T cell activation by binding to PD-1, contributing to an immunosuppressive tumor microenvironment. Furthermore, regulatory T cells (Tregs) may suppress immune responses in DCIS through the expression of negative co-stimulatory molecules, such as PD-1, PD-L1, and cytotoxic T-lymphocyte associated protein 4 (CTLA-4) [78,89]. TIGIT is often upregulated on exhausted CD8+ T cells and Tregs in DCIS and, together with co-expressed PD-1 on TILs, may serve as a marker of suppressed immune activity and progression to IDC [78].

The prognostic significance of stromal TILs in DCIS remains inconsistent. While increased overall TIL density has been associated with worse DFS, a higher risk of ipsilateral recurrence, and death from subsequent breast cancer, specific TIL subtypes show divergent prognostic implications [82,87,90,91,92]. A greater presence of CD3+CD8− T cells is linked to better outcomes, whereas elevated levels of CD4+FOXP3+ Tregs and a higher CD4+/CD8+ ratio correlate with worse prognosis and ipsilateral invasive recurrence [82,84]. In contrast, increased numbers of CD4+, CD3+, and CD8+ TILs—particularly intratumoral CD4+ and stromal CD8+ infiltration—have been associated with improved prognosis [93]. Higher B cell infiltration has been linked to shorter DFS [79]. On the other hand, Almekinders et al. found no clear correlation between TIL presence in DCIS and subsequent invasive breast cancer [83]. Compared to DCIS, invasive ductal carcinoma generally exhibits a higher infiltration of TILs, with CD4+ Tregs predominating, whereas CD8+ T cells are more frequent in DCIS [93,94]. Some studies, however, report the opposite trend [86]. Notably, the close proximity of T cells to tumor cells appears to be a favorable prognostic factor. DCIS cases with lower myoepithelial continuity scores tend to have better outcomes, suggesting that gaps in the MEC layer may facilitate cytotoxic T-cell interaction with tumor cells [84,95]. This aligns with findings that increased stromal CD8+ T-cell infiltration in high-grade DCIS is associated with a spontaneous healing process [96]. While the overall prognostic significance of TILs remains inconsistent, immune suppression and exhaustion—marked by increased Tregs, PD-1, TIGIT, and PD-L1—are key immunological features associated with disease progression and potential transition to IDC.

Natural killer (NK) cells are key effectors of the innate immune system, playing a vital role in immune surveillance and the elimination of tumor cells. Unlike T cells, NK cells can recognize and destroy malignant cells without prior antigen sensitization, acting rapidly through direct cytotoxicity and modulation of the immune response [97]. The presence of intratumoral NK cells has been positively correlated with better prognosis in several cancers [98,99,100]. The expression of specific surface genes on NK cells—particularly those involved in interactions with target cells such as tumor cells or dendritic cells (DCs)—correlates with favorable prognosis in BC patients [101]. However, tumor-associated NK cells also exert pro-angiogenic functions by secreting factors like VEGF and angiogenin [102]. VEGF not only promotes tumor angiogenesis but also contributes to immunosuppression by supporting the proliferation of immunosuppressive cell populations, impairing T-cell recruitment, and enhancing T-cell exhaustion [103]. Interestingly, the balance between the pro- and anti-tumor roles of NK cells appears to vary among BC subtypes. A strong NK cell presence is associated with a favorable prognosis in ER-positive and HER2-positive BC, whereas in TNBC, NK cell infiltration correlates with poor clinical outcomes [104]. To date, the role of NK cells in DCIS remains insufficiently characterized. One study demonstrated decreased expression of NK cell activation receptors in IDC compared to DCIS [105]. In contrast, another study reported no significant differences in the expression of NK cell-associated genes between DCIS and IDC. However, both DCIS and IDC exhibited significantly elevated expression levels compared to normal breast tissue [78]. Given the established role of NK cells in tumor surveillance, it is highly likely that they play a significant role in DCIS; however, this assumption requires further investigation.

Similarly, limited information is available regarding the role of other innate-like effector cells, such as NKT-like cells and γδ (gamma delta) T cells, in the development and progression of DCIS. NKT-like cells are defined by the co-expression of CD3, a T-cell marker, and CD56, typically associated with NK cells [106]. Due to their ability to mediate cytotoxicity and produce a broad range of cytokines, NKT-like cells are presumed to contribute to the immune-mediated elimination of malignant or infected cells. Indeed, a reduced frequency of NKT-like cells has been correlated with poorer prognosis in various cancer types [107]. The role of γδ T cells in breast cancer remains controversial. While one study reported that γδ T-cell infiltration was associated with improved prognosis across all breast cancer subtypes except TNBC [108], another found the opposite, linking high γδ T-cell presence with better outcomes, specifically in TNBC patients [109]. Additionally, IL-17-producing γδ T cells have been shown to promote tumor progression in murine models of breast cancer [110]. The density of γδ T cells in IDC has been reported to be significantly higher compared to both DCIS and normal breast tissue [78]. These findings may indicate that γδ T cells acquire pro-tumorigenic properties as breast cancer progresses. Overall, there is a notable lack of studies focusing on these less common immune cell populations in the context of DCIS, highlighting an important gap and opportunity for future research.

### 6.2. Fibroblasts

Fibroblasts are found in the stroma of breast tissue, mainly around ductal structures. At various stages of mammary gland development and pregnancy, they contribute to structural integrity by secreting matrix proteins that promote epithelial growth and differentiation. In addition to producing extracellular matrix (ECM) components, fibroblasts also release proteases, such as matrix metalloproteinase-3 (MMP3), to support stromal remodeling during branching morphogenesis [111]. Most opinions suggest that fibroblasts originate from primitive mesenchymal cells, whereas cancer-associated fibroblasts (CAFs) arise from activated fibroblasts within local tissues [112]. Additionally, epithelial cells in the tumor microenvironment (TME) can differentiate into CAFs through EMT [113]. Meanwhile, multiple studies indicate that a fraction of CAFs originate from mesenchymal stem cells (MSCs) [114]. CAFs play a crucial role in promoting cancer invasiveness and progression through multiple mechanisms. Fibroblasts produce proteases, such as MMPs, which degrade the extracellular matrix, leading to stromal reorganization and the release of growth factors [111]. Beyond ECM remodeling, CAFs are capable of contributing to DCIS invasion through collagen reorganization and secreting pro-tumorigenic cytokines and growth factors, such as interleukin-6 (IL-6), CXCL1, and vascular endothelial growth factor (VEGF), which support cancer cell proliferation, angiogenesis, and immune evasion [115,116,117,118]. To conclude, fibroblasts, particularly CAFs, are strongly associated with tumor progression in breast cancer, including the transition from DCIS to invasive disease.

### 6.3. Collagen

The ECM and its primary component, collagen, play a crucial role in various biological processes within normal tissues. In cancer, collagen is abnormally produced by CAFs, which interact with tumor cells to promote their proliferation, migration, and differentiation, ultimately driving cancer development and progression [119,120]. In breast cancer, the mammary gland undergoes progressive stiffening and becomes enriched in collagen compared to healthy breast tissue, largely due to aberrant matrix remodeling driven by CAFs. Tissue stiffness is closely linked to cancer grade, necrosis, and recurrence [121,122]. Fibrillar collagens like type I (COL1A1) and type III (COL3A1) are key in invasion and metastasis [123]. High COL1A1 levels correlate with worse survival in ER-positive breast cancer. Studies show that COL1A1 and COL11A1 are upregulated in breast cancer compared to normal tissue, making them potential markers for distinguishing between the two [124]. In contrast, COL3A1 is associated with triple-negative breast cancer (TNBC) and appears to promote invasiveness in this type of DCIS [125]. Additionally, high stromal COL11A1 expression was linked to lower recurrence-free survival, specifically for invasive breast cancer recurrence in the pure DCIS subset [126]. In summary, collagen facilitates tumor invasion through structural remodeling and biochemical signaling, acting as an active driver rather than a passive scaffold in breast cancer progression.

### 6.4. Macrophages

Finally, macrophages, a key component of the TME, reside in the stroma and gradually differentiate into tumor-associated macrophages (TAMs) [127]. During mammary gland development, they support tissue patterning, branching morphogenesis, and vascular growth [128]. Hypoxic conditions, growth factors, and immunosuppressive cytokines in the TME drive TAMs to acquire trophic macrophage characteristics [127]. Traditionally, macrophage subpopulations are classified as classically activated (M1), with proinflammatory and anti-tumor functions, or alternatively activated (M2), which suppress inflammation and promote tissue repair [129]. TAMs facilitate tumor progression by supporting angiogenesis, tumor migration, and invasion, as well as by remodeling the extracellular matrix and breaking down the basement membrane through the production of proteolytic enzymes and MMPs [130,131,132]. Additionally, TAMs suppress anti-tumor T-cell responses, enabling immune evasion and tumor growth by secreting anti-inflammatory cytokines (IL-10, TGF-β, PGE2) and inhibiting T-cell activation through checkpoint pathways (PD-1, CTLA-4) [133,134,135]. Taken together, these findings indicate that TAMs are closely linked to the progression of DCIS.

## 7. Epigenetics

Epigenetic modifications—including DNA methylation, histone modifications, and noncoding RNA regulation—are fundamental for imprinting and tissue-specific development [136]. However, their dysregulation can lead to global signaling alterations and drive pathological phenotypes. Understanding these epigenetic pathways and the molecular mechanisms that regulate them seems to be crucial for unraveling the pathology of DCIS progression [137].

DNA methylation plays a significant role in early tumorigenesis by silencing tumor suppressor genes and influencing the tumor microenvironment. Studies indicate that methylation changes occur early in the progression, primarily during the transition from normal tissue to DCIS, with genes like *HOXA10* and *SFRP1* being differentially methylated between DCIS and IDC [138,139,140,141]. Genome methylation studies revealed genes with differential methylation between DCIS and IDC patients with strong enrichment of homebox genes and polycomb repressive complex 2 (PRC2) targets [142,143,144]. On the other hand, another study indicated four other genes (*CPA1*, *CUL7*, *LRRTM2*, and *POU2AF1*) with increasing methylation from normal breast tissue through DCIS to IDC, suggesting their role in cancer progression [145]. In addition, prognostic signature based on the methylation levels of 18 CpG sites is linked to survival outcomes in breast cancer patients with invasive tumors, as well as those with DCIS and mixed DCIS-invasive lesions [145].

Histone modifications further regulate gene expression by altering chromatin accessibility [146]. Enhancer of zeste homolog 2 (EZH2), a PRC2 component, is increasingly expressed from normal tissue to IDC, indicating its role in tumor aggressiveness [147,148]. Similarly, histone deacetylases (HDACs) and lysine-specific demethylase 1 (LSD1) influence cancer progression by modifying chromatin structure. As the tumor advances, LSD1 expression increases while HDAC levels decrease, supporting the hypothesis that epigenetic deregulation is an early driver of cancer development [149,150].

Noncoding RNAs, particularly microRNAs (miRNAs) and long noncoding RNAs (lncRNAs), contribute to breast cancer progression by modulating gene expression and chromatin remodeling [151]. Dysregulated miRNAs influence tumorigenic pathways, including differentiation and metastasis, and variations in miRNA expression can distinguish breast cancer subtypes [152,153]. Recent studies have highlighted the miR-200 family and miR-205 as crucial regulators of EMT and maintainers of the epithelial phenotype [154]. In addition, miR-206 suppresses the growth and metastatic potential of breast cancer stem cells [155]. Similarly, lncRNAs play a key role in breast cancer progression. For instance, HOTAIR enhances invasive properties by interacting with epigenetic machinery [143,156]. Another lncRNA, BC200, is significantly overexpressed in high-grade DCIS and IDC compared to low-grade DCIS, suggesting its potential as a prognostic factor [157].

Together, these findings emphasize that epigenetic alterations are not only early events in breast cancer development but also potential drivers of DCIS progression, offering promising targets for diagnostic and therapeutic strategies.

## 8. Current Approach

Current strategies in the management of DCIS are evolving as research continues to refine risk stratification and treatment approaches. While DCIS is well-studied, the vast amount of available data remains difficult to standardize and integrate into clinical practice, leading to ongoing debates about overtreatment versus undertreatment [158]. To address this, clinical trials such as LORIS, COMET, and LORD are investigating active surveillance as an alternative to surgery for low-risk DCIS, aiming to identify patients who may safely avoid aggressive treatment [159,160,161]. Early results from COMET indicate that, among appropriately selected patients, active surveillance with endocrine therapy may be a safe and feasible approach, with no significant difference in short-term invasive cancer rates compared to immediate surgery [162]. However, several limitations of the study have been noted, including the reliance on diagnosis at the time of biopsy, where some cases with an invasive component may have been missed and subsequently misclassified as DCIS progression [163].

In parallel, molecular assays like Oncotype DX and DCISionRT are being used to assess recurrence risk and guide personalized treatment decisions [14]. Oncotype DX analyzes the expression of 12 genes to generate a DCIS score, which helps predict the likelihood of local recurrence and the potential benefit of radiation therapy after surgery. DCISionRT, on the other hand, integrates six molecular markers with clinical and pathological factors to provide a Decision Score, stratifying patients into low- or high-risk categories for recurrence and guiding the need for radiation or endocrine therapy. Both tests aim to personalize treatment, helping clinicians avoid overtreatment in low-risk cases while identifying those who may benefit from more aggressive management [164]. However, their global use is still limited due to the continued dominance of traditional approaches.

Despite these advancements, integrating the expanding body of knowledge into standardized and widely accepted clinical guidelines remains a challenge, underscoring the need for further research and innovative tools or technologies to uncover new perspectives.

## 9. Future Perspectives

Spatial techniques in cancer research have revolutionized our understanding of tumor heterogeneity, microenvironment interactions, and disease progression by enabling the precise localization of molecular information within tissue architecture [165,166]. The most comprehensive insights into cancer biology come from integrating genomic, transcriptomic, and proteomic data within a spatial framework [167]. However, even when used individually, these techniques provide valuable information. Among these techniques, spatial transcriptomics has recently emerged as a powerful tool, enabling researchers to map gene expression patterns across histological sections while preserving spatial context. Depending on the platform, it allows for the localization of thousands of transcripts—or even the entire transcriptome—within the analyzed tissue [165].

Tumor behavior is now being studied at the cellular level in its native tissue context, revealing microenvironment interactions that differ between the tumor core and its leading edge, offering key insights into invasion and metastasis [168,169]. Spatial analysis also helps map the progression from precancerous to malignant lesions, paving the way for early intervention [170]. Additionally, the role of the immune microenvironment is becoming clearer, exposing how cancer evades detection through immune suppression or T cell depletion [171].

Although spatial transcriptomics is a relatively new technique, continuously advancing each year with expanded capabilities, it has already been applied to nearly all solid tumors, significantly enhancing our understanding of specific cancer types [171,172,173,174,175]. It has also proved valuable in breast cancer research, where intratumoral heterogeneity plays a crucial role in treatment response and prognosis [176,177,178,179]. In DCIS studies, this approach is essential for distinguishing indolent cases from those with aggressive potential. By integrating spatial transcriptomics with histopathological features, researchers can identify distinct cellular populations, uncover novel biomarkers, and gain insights into tumor–stroma interactions that drive disease progression [180,181,182].

Various commercially available platforms support these investigations, broadly categorized by data acquisition methods. Imaging-based approaches include MERFISH (Vizgen) and Xenium (10x Genomics), while sequencing-based platforms such as Visium and Visium HD (10x Genomics), Stereo-seq (STOmics), and the GeoMx Digital Spatial Profiler (NanoString) offer high-resolution transcriptomic mapping [165]. These advancements not only refine our molecular understanding of breast cancer and DCIS but also open new possibilities for personalized medicine by identifying spatially defined therapeutic targets, drivers of progression, and resistance mechanisms. Its application in DCIS research holds promise for distinguishing aggressive cases from indolent ones, potentially guiding clinical decision-making.

## 10. Conclusions

DCIS is a complex and heterogeneous form of pre-invasive breast cancer that presents substantial challenges for clinical decision-making. As highlighted throughout this review, understanding which cases are likely to progress to invasive disease remains critical for optimizing patient care. Spatial transcriptomics, as an emerging and rapidly evolving technology, offers transformative potential in this regard. By enabling precise mapping of gene expression within native tissue architecture, spatial techniques have the potential to uncover previously unrecognized heterogeneity, reveal key tumor–microenvironment interactions, and identify novel biomarkers of disease progression. The integration of spatial data with histopathological and molecular features not only deepens our understanding of DCIS biology but also paves the way for more personalized, evidence-based therapeutic strategies. As spatial platforms continue to evolve, they are poised to become indispensable tools in the quest to balance effective treatment with the minimization of overtreatment in breast cancer care.

## Abbreviations

The following abbreviations are used in this manuscript:

BM	Basement membrane
CAFs	Cancer-associated fibroblasts
CK14	Cytokeratin 14
COL1A1	Collagen like type I
CTLA-4	Cytotoxic T-lymphocyte associated protein 4
CXCL14	Chemokine (C-X-C motif) ligand 14
DCIS	Ductal carcinoma in situ
DCIS-Mi	DCIS with microinvasion
DCs	Dendritic cells
DFS	Disease-free survival
ECM	Extracellular matrix
EMT	Epithelial–mesenchymal transition
ER	Estrogen receptor
EZH2	Enhancer of zeste homolog 2
HDAC	Histone deacetylases
HER2	Human epidermal growth factor 2
HR	Hormone receptor
IDC	Invasive ductal carcinoma
IL-6	Interleukin-6
lncRNA	Long noncoding RNA
LSD1	Lysine-specific demethylase 1
MECs	Myoepithelial cells
miRNA	MicroRNA
MMP3	Matrix metalloproteinase-3
MSCs	Mesenchymal stem cells
NK	Natural killer
PD-1	Programmed cell death receptor 1
PD-L1	Programmed death-ligand 1
PR	Progesterone receptor
PRC2	Polycomb repressive complex 2
SMMHC	Smooth muscle myosin heavy chain
TAMs	Tumor-associated macrophages
TCF7	T-cell factor 7
TGFβ1	Transforming growth factor beta 1
TIGIT	T cell immunoreceptor with immunoglobulin and ITIM domain
TILs	Tumor-infiltrating lymphocytes
TME	Tumor microenvironment
TNBC	Triple-negative breast cancer
Tregs	Regulatory T cells
VEGF	Vascular endothelial growth factor
α-SMA	α-Smooth muscle actin
